# Optimization of a Rabbit Dry Eye Model Induced by Topical Instillation of Benzalkonium Chloride

**DOI:** 10.1155/2020/7204951

**Published:** 2020-05-30

**Authors:** Carlos Carpena-Torres, Jesús Pintor, María Jesús Pérez de Lara, Fernando Huete-Toral, Almudena Crooke, Cristina Pastrana, Gonzalo Carracedo

**Affiliations:** ^1^Department of Optometry and Vision, Faculty of Optics and Optometry, Universidad Complutense de Madrid, Madrid, Spain; ^2^Department of Biochemistry and Molecular Biology, Faculty of Optics and Optometry, Universidad Complutense de Madrid, Madrid, Spain

## Abstract

**Purpose:**

To optimize a rabbit dry eye model induced by topical instillation of benzalkonium chloride (BAC), reduce the days of instillation of the original model by increasing the concentration of BAC from 0.1% to 0.2%.

**Materials and Methods:**

An experimental, prospective, and randomized study was performed on 10 male New Zealand white rabbits, divided into two groups, considering both eyes: 5 rabbits as control (*n* = 10) and 5 rabbits with 0.2% BAC treatment (*n* = 10). Saline solution (control) and 0.2% BAC were instilled for 5 consecutive days, twice daily. Tear secretion with and without anesthesia, tear breakup time, tear osmolarity, corneal staining, conjunctival hyperemia, density of goblet cells, height of mucin cloud, and transcript levels of IL-6 were measured before and after the treatment.

**Results:**

After the instillation of 0.2% BAC for 5 consecutive days, there was a significant increase in tear secretion without anesthesia (*P* < 0.001), corneal staining (*P* < 0.001), conjunctival hyperemia (*P* < 0.001), and levels of IL-6 mRNA (*P*=0.005) compared to the control group. Conversely, there was a decrease in tear secretion with anesthesia (*P* < 0.001), tear breakup time (*P*=0.007), tear osmolarity (*P* < 0.001), density of goblet cells (*P* < 0.001), and height of mucin cloud (*P* < 0.001).

**Conclusions:**

The topical instillation of 0.2% BAC for 5 consecutive days, twice daily, was a proper procedure to induce a rabbit dry eye model, reducing the number of days of instillation compared to the original model (14 days).

## 1. Introduction

In 2017, the TFOS DEWS II defined the dry eye as “*a multifactorial disease of the ocular surface characterized by a loss of homeostasis of the tear film, and accompanied by ocular symptoms, in which tear film instability and hyperosmolarity, ocular surface inflammation and damage, and neurosensory abnormalities play etiological roles*” [1]. Dry eye affects the quality of life of a large part of the world population [2], between 5% and 50%, depending on geographic location and methodology used to diagnose the disease [3].

The discovery of new diagnostic biomarkers and treatments for dry eye requires the use of animal models. Based on the etiology and classification of dry eye, different animal models have been developed over the years [4, 5]. In rabbits, dry eye has been induced by many procedures such as dacryoadenectomy [6], female and male castration [7, 8], desiccating stress [9], induced autoimmune dacryoadenitis [10], injection of the lacrimal gland with concanavalin A [11], injury of the drainage duct [12], nerve denervation [13], and topical instillation of atropine [14] or benzalkonium chloride (BAC) [15]. The current publication is focused on the topical instillation of BAC.

BAC is a preservative agent, which is incorporated into the formulation of eye drops and contact lens solutions. It is frequently used in glaucoma medication. BAC could produce undesirable side effects over the ocular surface of these patients because of its toxicity and irritancy [16, 17]. The concentrations of BAC used in commercial formulations are between 0.005% and 0.02%, which lyse the membranes of corneal epithelium cells [18]. BAC also produces damage in the structure of tear film constituents [19], corneal innervation [20], and conjunctival cells [21]. Thus, the topical instillation of BAC has been used to develop dry eye models in rabbits [15] and mice [22].

Animal models based on the topical administration of BAC allow the evaluation of different dry eye markers related to tear quality, ocular surface damage, and inflammation. In rabbits, several studies used this model to evaluate the efficacy of different eye drops containing hyaluronic acid [23, 24], povidone [23], epigallocatechin gallate [24, 25], virally inactivated serum [26], carboxymethyl pullulan [27], or cyclosporine A [28]. This model was also used to evaluate new treatments using drug delivery systems such as nanoparticles modified with anti-inflammatories [29] or epigallocatechin gallate/hyaluronic acid [30] and cyclosporine A-eluting contact lenses [31]. Other authors studied the efficacy of the intracanalicular injection of a chitosan-based hydrogel for dry eye treatment [32] and the influence of dry eye in the glaucoma filtration surgery [33].

The original rabbit dry eye model induced by topical instillation of BAC was developed by Xiong et al. [15] who proposed the instillation of 0.1% BAC for 2 consecutive weeks, twice daily. Additionally, they evaluated higher concentrations of BAC (0.2%, 0.3%, 0.5%, and 1%) which were not safe, but they did not report results about this fact. Other authors used this model induced through the instillation of 0.1% BAC for three [33] and four [24, 26, 30] consecutive weeks. In mice, the original dry eye model was developed by Lin et al. [22] who instilled 0.2% BAC for 1 week, twice daily, reducing the time by half, compared to the same model in rabbits. This mouse dry eye model has been used effectively by other authors [34, 35].

The current study is focused on the idea of reducing the time of instillation of BAC necessary to induce the rabbit dry eye model by increasing the concentration of BAC from 0.1% to 0.2%.

Based on the previous studies, the purpose of the current study was to optimize the original rabbit dry eye model induced by topical instillation of BAC [15], reducing the number of days of instillation. To that end, 0.2% BAC was instilled for 5 consecutive days, twice daily (except the last day). Signs of dry eye related to tear quality, ocular surface damage, and inflammation were evaluated.

## 2. Materials and Methods

### 2.1. Design of the Study

An experimental, prospective, and randomized study was performed in compliance with the ARVO Statement for the Use of Animals in Ophthalmic and Vision Research and with the Directive 2010/63/EU of the European Parliament and of the Council on the protection of animals used for scientific purposes.

All the trials were performed before (pre) and after (post) the instillation of saline solution (Avizor, Madrid, Spain) as control and 0.2% BAC (Merck, Darmstadt, Germany). Two instillations of 35 *μ*l per day for 5 consecutive days were performed: one in the morning (10 a.m.) and another in the evening (6 p.m.), from Monday to Friday. On the last day, only one instillation during the morning was done and the measurements were taken after 1 hour. In total, nine instillations per rabbit were performed. The concentration of BAC and the number of days of instillation were selected based on the results of a previous pilot study. The order of the trials was as follows: tear osmolarity, tear secretion without anesthesia, slit-lamp examination, conjunctival cytology, and tear secretion with topical anesthesia. Topical anesthesia to assess tear secretion was instilled once the rest of the measurements were done.

### 2.2. Animals

Ten male New Zealand white rabbits were used in the study, considering both eyes (*n* eyes = 20), randomly divided into two groups: 5 rabbits as control (*n* = 10) and 5 rabbits with 0.2% BAC (*n* = 10). The weight of the rabbits was between 2.0 and 2.5 kg. The rabbits were kept in cages with free access to food and water. The rabbits were kept under controlled 12 h light-dark cycles, a temperature of 18°C, and a humidity of 30%. Before experimentation, the rabbits were kept in the cages for 7 days to get them used to their new housing conditions.

### 2.3. Pilot Study

A pilot study was performed to establish the final concentration of BAC (0.2%) and the number of days of instillation (5 days). The presence of undesirable signs such as corneal ulcer, neovascularization, and scarring was evaluated over time with the slit-lamp VX75 (Luneau Technology, Chartres, France). Eight rabbits were used: 2 rabbits received saline solution, 2 rabbits received 0.1% BAC, 2 rabbits received 0.2% BAC, and 2 rabbits received 0.3% BAC. All the treatments were instilled for 2 consecutive weeks, twice daily, doing two washout periods during the weekends.

**Figure 1:**
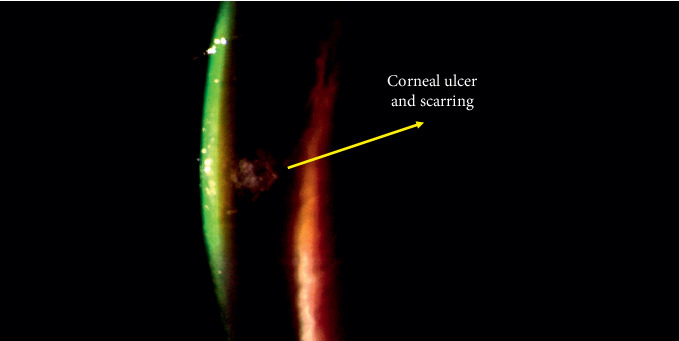
Representative image of corneal ulcer and scarring produced by the topical instillation of 0.3% BAC after 10 instillations. These signs were similar after the instillation of 0.2% BAC, but they appeared later (after 15 instillations). The lack of transparency of the cornea can be observed, due to a stromal ulcer by using the technique of indirect illumination with a slit lamp.

Table 1 specifies the presence of corneal ulcer, neovascularization, and scarring over time with the different concentrations of BAC, while Figure 1 shows a representative image of the corneal ulcer and scarring produced in the rabbits.

### 2.4. Tear Secretion, Breakup Time, and Osmolarity

Tear secretion was measured using Schirmer's strip (Lenticon, Madrid, Spain) for 5 min. Each millimeter of the strip soaked with tear corresponds to 1 *μ*l. The paper strip was positioned in the inferior temporal eyelid, and the rabbits' eyes were closed during the measurements to avoid reflex blinking. The measurements were taken with and without topical anesthesia. For measurements with anesthesia, two drops of a commercial eye drop containing 5 mg/ml tetracaine hydrochloride and 0.5 mg/ml naphazoline hydrochloride (Alcon Cusí, Barcelona, Spain) were instilled with a difference of 5 min between instillations. The measurements were taken 5 min after the last instillation.

Tear breakup time was measured during the slit-lamp examination. For its evaluation, 5 *μ*L of commercial 2% fluorescein sodium (Alcon Cusi, Barcelona, Spain) was instilled over the ocular surface. The measurements were taken with a timer after manually force two consecutive blinks to the rabbits.

Tear osmolarity was measured with the medical device TearLab (TearLab Corporation, San Diego, California, United States), an osmometer which analyses the electrical impedance of 50 nL of the tear film. All the measurements were taken in the same room at 18°C. Both eyes were measured consecutively, first the right eye and then the left one.

### 2.5. Slit-Lamp Examination

The signs of ocular surface damage were evaluated with the slit-lamp VX75 by using the same commercial 2% fluorescein sodium as for measuring tear breakup time. The Efron Grading Scales were used to quantify the severity of corneal staining and conjunctival hyperemia, grading the signs as normal (0), trace (1), mild (2), moderate (3), and severe (4) [36].

Additionally, corneal ulcer, neovascularization, and scarring were assessed to confirm the safety of the instillation of 0.2% BAC.

### 2.6. Conjunctival Cytology

Conjunctival cytology was done to collect the superficial conjunctival cells with the medical device EYEPRIM (Opia Technologies, Paris, France). Cytology of the superior and inferior quadrants of the bulbar conjunctiva of each eye was taken.

**Figure 2:**
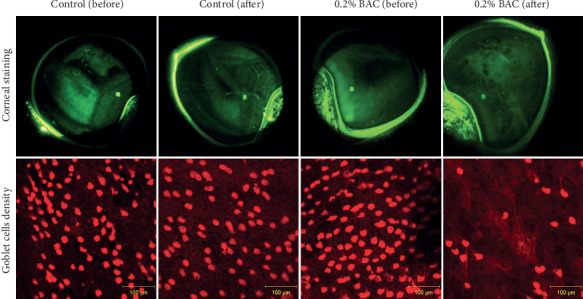
Representative images of ocular surface damage in terms of corneal staining and density of goblet cells before and after the instillation of saline solution (control) and 0.2% benzalkonium chloride (BAC) for 5 days. After the instillation of 0.2% BAC, an increase is observed in corneal superficial punctate and a decrease in the density of goblet cells (brightest cells).

Superior cytology was fixed in 96% ethanol for 24 hours at 4°C. Then, the samples were stained with the hematoxylin-periodic acid Schiff (PAS) procedure. The goblet cells of the conjunctiva were evaluated by using the confocal microscopy Axiovert 200 M (Zeiss, Oberkochen, Germany). The density of goblet cells (Figure 2) was quantified in 5 different regions of each sample. The height of mucin cloud, including the cell thickness, was quantified in 15 different cells of each sample. Both sample visualization and their measurements were done by using the LSM Image Browser software (Zeiss). The hematoxylin of the samples was excited by a wavelength of 488 nm, and its signal was filtered for a range between 505 and 530 nm. On the other hand, the PAS was excited by a wavelength of 543 nm, and its signal was filtered for 560 nm. The *Z*-stack to visualize the three-dimensional cells was done for a pupil size of 180 *μ*m and a stack interval of 0.25 *μ*m. All these procedures were done according to Peral and Pintor [37].

Inferior cytology was fixed in RNAlater (Thermo Fisher Scientific, Waltham, Massachusetts, USA) for 24 hours at 4°C. Then, RNAlater was removed, and the samples were stored at −80°C for their posterior processing.

### 2.7. RNA Isolation, cDNA Synthesis, and Quantitative PCR

The purpose of the following procedures was to quantify the levels of interleukin 6 (IL-6) mRNA in conjunctival cells.

Total RNA was isolated from inferior cytology with the QIAshredder and RNeasy Mini Kit (Qiagen, Madrid, Spain) following the manufacturer's instructions.

First-strand cDNA synthesis was performed from 22 *μ*L of total RNA, using High Capacity cDNA Reverse Transcription Kit and random hexamer primers (Thermo Fisher Scientific). Quantitative PCR (qPCR) was performed in triplicate using cDNA, the QuantiTect SYBR Green Kit (Qiagen), and IL-6 specific primers (5′-GCCTCACAAACTTCCTGGAG-3′/5′-GATGGTGTGTTCTGACCGTG-3′) on a QuantStudio 3 system (Thermo Fisher Scientific). The thermal cycler program was 15 min at 95°C, followed by 40 cycles of 15 s at 94°C, 30 s at 55°C, and 34 s at 72°C (data collection step). Nontemplate and nonreverse transcribed controls were included in all the experiments.

Analysis of the melting curves confirmed the specificity of PCR and the absence of primer-dimers. The hypoxanthine-guanine phosphoribosyltransferase 1 (HPRT1) gene (5′-CTGGCAAAACAATGCAGACCT-3′/5′-GTCCTTTTCACCAGCAGGCTT-3′) was used as an internal control to normalize mRNA relative expression, after its validation for qPCR. Validation of the internal control gene and qPCR data analysis were performed by the 2^−ΔCt^ and 2^−Δ∆Ct^ method, respectively, once confirmed that the amplification efficiency of IL-6/HPRT1 primer pairs was similar and close to a value of 2.

### 2.8. Statistical Analysis

Statistical analysis was performed using the SPSS Statistics 23 software (IBM, Chicago, Illinois, United States). The normality of each distribution was assessed using the Shapiro–Wilk test. The statistical comparison between the baseline measurements (PRE) and after the instillation of the treatments (POST) was done using Student's *t*-test for paired samples, in case of normal distributions, and the Wilcoxon signed-rank test, in case of nonnormal distributions. Additionally, the statistical comparison between the effect of each treatment was done using Student's *t*-test for independent samples, in case of normal distributions, and the Mann–Whitney *U* test, in case of nonnormal distributions. A statistical significance of 95% was established (*P*=0.05) in all the tests.

The studied parameters were tear secretion without anesthesia, tear secretion with anesthesia, tear breakup time, tear osmolarity, corneal staining, conjunctival hyperemia, density of goblet cells, height of mucin cloud, and levels of IL-6 mRNA. Results are shown as mean ± SD.

## 3. Results

The instillation of 0.2% BAC for 5 consecutive days did not produce corneal ulcer, neovascularization, or scarring in any rabbit, confirming the safety of this procedure. Figure 2 shows the ocular surface damage produced by the instillation of 0.2% BAC in terms of corneal staining and density of goblet cells.

Table 2 summarizes the values of all the parameters under study before and after the instillation of 0.2% BAC and saline solution (control) for 5 consecutive days.

### 3.1. Tear Secretion, Breakup Time, and Osmolarity

**Figure 3:**
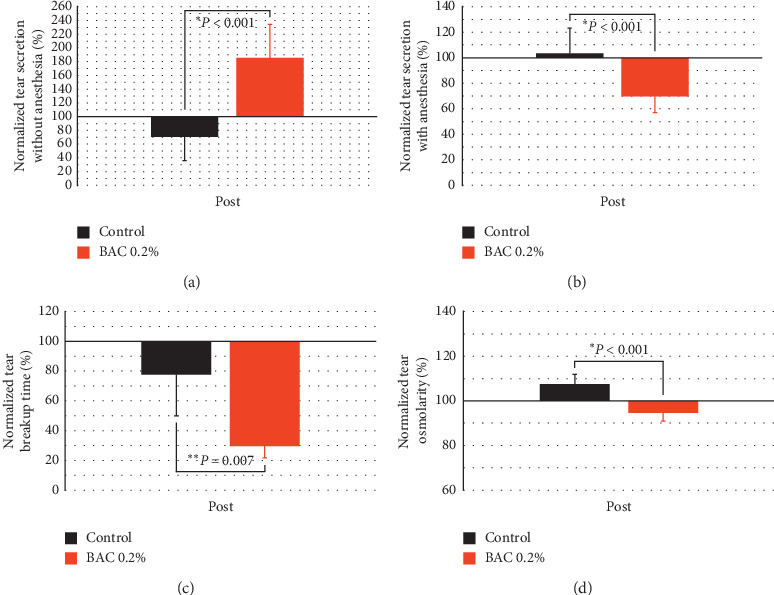
Normalized effect on tear parameters after the instillation of saline solution (control) and 0.2% benzalkonium chloride (BAC) for 5 days. Tear secretion without (a) and with anesthesia (b), tear breakup time (c), and tear osmolarity (d). The instillation of 0.2% BAC decreased tear secretion with anesthesia, tear breakup time, and tear osmolarity compared to the control group, while tear secretion without anesthesia was increased. Values higher and lower than 100% represent an increase or decrease, respectively, compared to their baseline. Statistical comparison was done between both groups. ^*∗*^*P* < 0.05, Student's *t*-test for independent samples. ^*∗∗*^*P* < 0.05, the Mann–Whitney *U* test.

To begin with the comparison between both groups, Figure 3 shows the normalized effect of both treatments on tear parameters (tear secretion, tear breakup time, and tear osmolarity). Concerning tear secretion, different results were found with and without topical anesthesia. Without anesthesia, it was found a statistical increase of 85.42 ± 22.51% after the instillation of 0.2% BAC compared to the control group that suffered a decrease of 29.41 ± 14.43% (*P* < 0.001; comparison between groups). Conversely, with anesthesia, there was a decrease of 30.30 ± 5.33% with 0.2% BAC compared to the control group that showed no statistical differences (*P* < 0.001).

About tear breakup time, both treatments showed a statistical decrease, but it was higher after the instillation of 0.2% BAC (70.47 ± 18.86%) compared to the control group (22.47 ± 8.01%) (*P*=0.007). Finally, there was a statistical decrease in tear osmolarity of 5.40 ± 0.21% after the instillation of 0.2% BAC compared to the control group (*P* < 0.001), which showed a statistical increase of 7.46 ± 0.30%.

### 3.2. Slit-Lamp Examination

**Figure 4:**
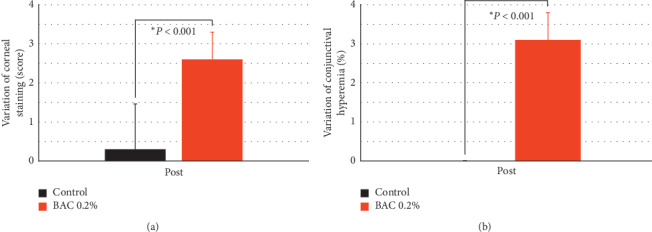
Variation of corneal staining (a) and conjunctival hyperemia (b) after the instillation of saline solution (control) and 0.2% benzalkonium chloride (BAC) for 5 days. The instillation of 0.2% BAC produced a deterioration of both variables compared to the control group. Positive values represent a deterioration compared to their baseline. Statistical comparison was made between both groups. ^*∗*^*P* < 0.05, the Mann–Whitney *U* test.

Figure 4 shows the effect of both treatments on corneal staining and conjunctival hyperemia. These values were not normalized in percentage since they are discrete variables. After the instillation of 0.2% BAC, there was a deterioration in the score of both corneal staining (2.60 ± 0.70) and conjunctival hyperemia (3.10 ± 0.74) compared to the control group (*P* < 0.001), where there were no statistical differences.

### 3.3. Conjunctival Cytology

**Figure 5:**
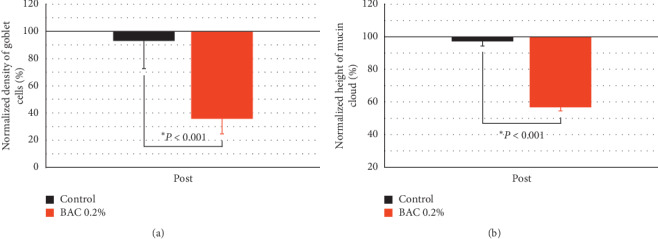
Normalized effect on goblet cells after the instillation of saline solution (control) and 0.2% benzalkonium chloride (BAC) for 5 days. Density of goblet cells (a) and height of mucin cloud (b). The instillation of 0.2% BAC deteriorated both the quantity and quality of these cells compared to the control group. Values higher and lower than 100% represent an increase or decrease, respectively, compared to their baseline. Statistical comparison was made between both groups. ^*∗*^*P* < 0.05, Student's *t*-test for independent samples.

Figure 5 shows the normalized effect of both treatments on the parameters related to goblet cells (density of goblet cells and height of mucin cloud). The instillation of 0.2% BAC produced a decrease of both the density of goblet cells (64.32 ± 19.92%) and height of mucin cloud (43.15 ± 1.72%) compared to the control group (*P* < 0.001) that showed no statistical differences.

### 3.4. Quantitative PCR

**Figure 6:**
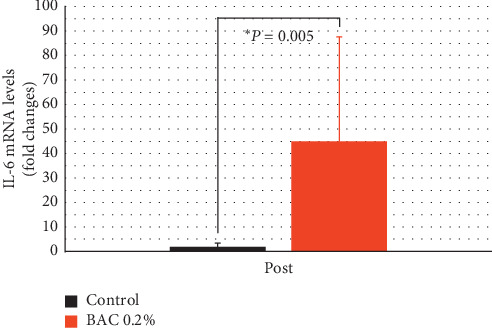
Interleukin 6 (IL-6) mRNA fold change in conjunctival cells after the instillation of saline solution (control) and 0.2% benzalkonium chloride (BAC) for 5 days. The instillation of 0.2% BAC increased the levels of this marker of ocular surface inflammation in the conjunctiva compared to the control group. Positive values represent an increase in this expression compared to their baseline. Statistical comparison was made between both groups. ^*∗*^*P* < 0.05, the Mann–Whitney *U* test.

Figure 6 shows the effect of both treatments on levels of IL-6 mRNA. The results were normalized against HPRT1 signal (internal control) and against levels of IL-6 mRNA in baseline conditions (PRE). In levels of IL-6 mRNA (fold change), there were no statistical changes in the control group and was an increase of 44.87 ± 42.70 with 0.2% BAC (*P*=0.005). Comparing the effect of both treatments, the instillation of 0.2% BAC produced a 24.52-fold increase.

## 4. Discussion

The current study reports on the possibility of reducing the time necessary to induce a rabbit dry eye model based on the topical instillation of BAC. It was possible to reduce this time from 14 days of the original model of Xiong et al. [15] to 5 days of the current one by increasing the concentration of BAC from 0.1% to 0.2%. The instillation of 0.2% BAC for 5 consecutive days, twice daily, deteriorated some signs of dry eye such as tear breakup time, fluorescein corneal staining, conjunctival hyperemia, density of goblet cells, height of mucin cloud, and levels of IL-6 mRNA. Conversely, this instillation increased tear secretion and decreased tear film osmolarity, which could be associated to the different factors discussed below.

In terms of safety, the instillation of 0.2% BAC for 5 consecutive days did not produce corneal ulcer or neovascularization. In our previous pilot study, it was observed that the instillation of 0.2% BAC produced corneal ulcer during the second week (see Table 1), accompanied by neovascularization and scarring. Xiong et al. [15] evaluated higher concentrations of BAC than 0.1% (0.2%, 0.3%, 0.5%, and 1%), different frequencies of instillation (1–4 times daily), and different lengths of treatment (1–4 weeks) to optimize their model. They established that higher concentrations of BAC than 0.1% caused corneal ulcer, neovascularization, and scarring over a period of 2 weeks. However, they did not report data about this fact, even during the first week of evaluation. Since our results did not show any undesirable effect over the ocular surface, the instillation of 0.2% BAC for 5 consecutive days could be considered a proper method to induce dry eye in rabbits. In agreement with our results, other authors instilled 0.2% BAC for 1 week to induce dry eye in mice [22, 34, 35]. It should be taken into consideration that the anatomy and physiology of rabbits and mice are not the same, but the cornea of mice is even thinner than the cornea of rabbits [38, 39].

From the safety perspective of this dry eye model, its success rate could be considered as 100% since none of the rabbits' eyes showed undesirable side effects after the instillation of 0.2% BAC for 5 consecutive days. Concerning its efficacy, 100% of the eyes suffered a deterioration of tear secretion with anesthesia, tear breakup time, corneal staining, conjunctival hyperemia, density of goblet cells, and height of mucin cloud, while 80% of these eyes increased their levels of IL-6 mRNA.

Concerning tear secretion, it should be noted that the effect of the instillation of 0.2% BAC was completely different with or without topical anesthesia. Without anesthesia, the 0.2% BAC produced an increase in tear secretion around 36% compared to the control group (see Figure 3(a)), the opposite effect that would be expected in an aqueous deficient dry eye [1]. This hypersecretion was associated with ocular irritation after the instillation of this compound [16, 17], which could be producing reflex lacrimation. Conversely, with anesthesia, there was a decrease of tear secretion as expected, around 34% compared to the control group (see Figure 3(b)). Our results with anesthesia would agree with different studies that found a similar decrease in tear secretion by using both topical [15] and general anesthesia [24, 26, 27, 30–33]. Therefore, the use of anesthesia would be necessary to evaluate tear secretion in this dry eye model. The values of tear secretion with anesthesia obtained by these mentioned dry eye models were between 3 and 10 *μ*L, performing Schirmer's test for 5 min [15, 24, 26, 27, 30, 31, 33].

As expected, tear breakup time was reduced after the instillation of 0.2% BAC around 48% compared to the control group (see Figure 3(c)). This severe instability of the tear film is part of the pathophysiology of dry eye, which comes from inflammation and damage of the ocular surface (see Figures 4–6) [40]. In our knowledge, only Choi et al. [31] evaluated tear breakup time using this model after the instillation of 0.1% BAC for 2 weeks. They found a reduction of around 73% compared to their control group. Directly comparing values of tear breakup time with other studies should be done carefully since these values depend on different factors such as both volume and concentration of instilled fluorescein [41].

Tear osmolarity was the other parameter whose results were not as expected in a dry eye model [1]. In the current study, the instillation of 0.2% BAC produced a decrease in tear osmolarity around 13% compared to the control group (see Figure 3(d)). No studies evaluating tear osmolarity in this dry eye model were found in the scientific literature. This decrease in tear osmolarity could be related to the increase in tear secretion without anesthesia, since osmolarity was also measured without it. An increase in the tear volume could dilute the ionic compounds present in tear, reducing its osmolarity. This theory is based on the negative correlation found between tear osmolarity and tear secretion (*r*=−0.791, *P* < 0.001) in the sample of this study. Additionally, Kim et al. [42] found a negative correlation between both parameters (*r*=−0.625, *P* < 0.001) in primary Sjögren's syndrome patients.

In the control group, it was observed that all the tear parameters, except tear secretion with anesthesia, were deteriorated after the instillation of saline solution for 5 days (see Table 2). Since saline solution should not produce any undesirable effects on the ocular surface, these changes would be associated with environmental factors.

In relation to ocular surface damage, all the rabbits reached the maximum score of corneal staining after the instillation of 0.2% BAC (see Table 2). This corneal damage was accompanied by conjunctival damage in terms of conjunctival hyperemia (see Figure 4(b)) and a reduction in density of goblet cells around 57% compared to the control group (see Figure 5(a)). Additionally, the functionality of these goblet cells was affected considering the reduction in their height of mucin cloud, around 40% compared to the control group (see Figure 5(b)). The severity of these markers suggests that the instillation of 0.2% BAC is a fast method to induce ocular surface damage. In the original rabbit dry eye model, Xiong et al. [15] found a lower corneal staining score after the instillation of 0.1% BAC for 2 weeks compared to the current study, but a higher loss of goblet cells. As expected, other authors found similar ocular surface damage [23–33]. Additionally, some of them reported a reduction in epithelial corneal thickness [24, 29, 30], which is another marker that could be evaluated in this animal model.

On the other hand, the high levels of IL-6 mRNA in conjunctival cells after the instillation of 0.2% BAC confirmed the presence of ocular surface inflammation (see Figure 6). It should be noted that the standard deviation in the 0.2% BAC treated group was similar to its mean variation. This was because two eyes showed lower levels of IL-6 mRNA than their baseline measurements. The rest of eyes showed a range of expression levels from 9.69 to 99.80 times higher than their baseline measurements. After the instillation of 0.1% BAC, some studies also found an increase in expression levels of IL-6 [24, 30, 31] and other molecular biomarkers in conjunctival cells such as IL-1*β* [30, 31, 33], IL-8 [24, 30], and TNF-*α* [24, 30, 31]. Other authors reported a decrease in functionality of goblet cells in terms of expression levels of MUC5AC [15, 29].

This rabbit dry eye model has been used to measure the efficacy of different eye drops and drug delivery systems for dry eye treatment [23–32]. Some studies applied their treatments while the BAC was being instilled [23, 27–29, 31] and other studies after the instillation of BAC, during the period of reversibility of the dry eye model [24–26, 30, 32]. Li et al. [43] studied the stability of the original model of Xiong et al. [15], establishing that the signs of dry eye were sustained between 2 and 3 weeks after the last instillation of 0.1% BAC. Taking into consideration that both alternatives were effective, it would be logical to apply the treatments while the BAC is being instilled, in order to save time in the experiments. However, a comparison between both alternatives should be considered for future studies, considering that if the dry eye is induced in a shorter time, the recovery of clinical signs could be different compared to the study of Li et al. [43].

The current study had some limitations that could be improved in future studies. Considering that tear osmolarity was decreased after the instillation of 0.2% and its negative correlation with tear secretion without anesthesia, topical anesthesia could be probably necessary to measure tear osmolarity in this dry eye model. Besides, the number of days of instillation could have been reduced even more if the rabbits had been evaluated every day during the pilot study.

## 5. Conclusions

In conclusion, the topical instillation of 0.2% BAC for 5 consecutive days, twice daily, was a proper procedure to induce a rabbit dry eye model, reducing the number of days of instillation compared to the original model (14 days) [15]. Additionally, it is emphasized that topical or general anesthesia must be used in future studies to evaluate tear secretion.

## Figures and Tables

**Table 1:** Results of the pilot study on the presence of corneal ulcer, neovascularization, and scarring after the instillation of saline solution (control) and the different concentrations of BAC (0.1%, 0.2%, and 0.3%).

Corneal sign	Group	Number of instillations			
		First week		Second week	
		5	10	15	20
Ulcer	Control	−	−	−	−
	0.1% BAC	−	−	−	−
	0.2% BAC	−	−	+	+
	0.3% BAC	−	+	+	+

Neovascularization	Control	−	−	−	−
	0.1% BAC	−	−	−	−
	0.2% BAC	−	−	+	+
	0.3% BAC	−	+	+	+

Scarring	Control	−	−	−	−
	0.1% BAC	−	−	−	−
	0.2% BAC	−	−	+	+
	0.3% BAC	−	+	+	+

The positive and negative signs indicate the presence or absence of these signs, respectively.

**Table 2:** Values of all the parameters under study before (pre) and after (post) the instillation of saline solution (control) and 0.2% benzalkonium chloride (BAC) for 5 days.

Parameter	Group	Mean ± SD		*P* value
		Pre	Post	
Tear secretion without anesthesia (*μ*L)	Control	11.90 ± 2.89	8.40 ± 4.12	0.032^*∗*^
	0.2% BAC	9.60 ± 3.13	17.80 ± 4.69	0.008^*∗*^
Tear secretion with anesthesia (*μ*L)	Control	5.80 ± 1.03	6.00 ± 1.15	0.414
	0.2% BAC	6.60 ± 0.52	4.60 ± 0.84	0.004^*∗∗*^
Tear breakup time (s)	Control	5.03 ± 1.82	3.90 ± 1.39	0.036^*∗∗*^
	0.2% BAC	4.30 ± 1.15	1.27 ± 0.34	0.005^*∗∗*^
Tear osmolarity (mOsm/L)	Control	309.70 ± 13.66	332.80 ± 13.49	0.008^*∗*^
	0.2% BAC	313.00 ± 12.75	296.10 ± 11.71	0.014^*∗*^
Corneal staining (score)	Control	1.40 ± 0.70	1.70 ± 0.95	0.405
	0.2% BAC	1.40 ± 0.70	4.00 ± 0.00	0.004^*∗∗*^
Conjunctival hyperemia (score)	Control	0.10 ± 0.32	0.10 ± 0.32	1.000
	0.2% BAC	0.30 ± 0.68	3.40 ± 0.52	0.004^*∗∗*^
Density of goblet cells (cells/mm^2^)	Control	683.57 ± 77.63	636.19 ± 140.20	0.304
	0.2% BAC	703.12 ± 75.76	250.89 ± 77.71	<0.001^*∗*^
Height of mucin cloud (*μ*m)	Control	27.48 ± 0.74	26.71 ± 0.79	0.469
	0.2% BAC	27.72 ± 0.54	15.76 ± 0.63	<0.001^*∗*^
Expression levels of IL-6 (fold change)	Control	0.020 ± 0.031	0.017 ± 0.008	0.770
	0.2% BAC	0.032 ± 0.063	0.322 ± 0.252	0.002^*∗∗*^

Statistical comparison was done between pre and postvalues. ^*∗*^*P* < 0.05, Student's *t*-test for paired samples. ^*∗∗*^*P* < 0.05, the Wilcoxon signed-rank test.

## Data Availability

The data used to support the findings of this study are available from the corresponding author upon request.
